# Deep convolutional neural network-based classification of cancer cells on cytological pleural effusion images

**DOI:** 10.1038/s41379-021-00987-4

**Published:** 2022-01-10

**Authors:** Xiaofeng Xie, Chi-Cheng Fu, Lei Lv, Qiuyi Ye, Yue Yu, Qu Fang, Liping Zhang, Likun Hou, Chunyan Wu

**Affiliations:** 1 Department of Pathology, Tongji University Affiliated Shanghai Pulmonary Hospital, Shanghai, China; 2 Shanghai Aitrox Technology Corporation Limited, Shanghai, China

**Keywords:** Cancer imaging, Translational research

## Abstract

Lung cancer is one of the leading causes of cancer-related death worldwide. Cytology plays an important role in the initial evaluation and diagnosis of patients with lung cancer. However, due to the subjectivity of cytopathologists and the region-dependent diagnostic levels, the low consistency of liquid-based cytological diagnosis results in certain proportions of misdiagnoses and missed diagnoses. In this study, we performed a weakly supervised deep learning method for the classification of benign and malignant cells in lung cytological images through a deep convolutional neural network (DCNN). A total of 404 cases of lung cancer cells in effusion cytology specimens from Shanghai Pulmonary Hospital were investigated, in which 266, 78, and 60 cases were used as the training, validation and test sets, respectively. The proposed method was evaluated on 60 whole-slide images (WSIs) of lung cancer pleural effusion specimens. This study showed that the method had an accuracy, sensitivity, and specificity respectively of 91.67%, 87.50% and 94.44% in classifying malignant and benign lesions (or normal). The area under the receiver operating characteristic (ROC) curve (AUC) was 0.9526 (95% confidence interval (CI): 0.9019–9.9909). In contrast, the average accuracies of senior and junior cytopathologists were 98.34% and 83.34%, respectively. The proposed deep learning method will be useful and may assist pathologists with different levels of experience in the diagnosis of cancer cells on cytological pleural effusion images in the future.

## Introduction

Lung cancer is one of the leading causes of cancer-related death worldwide^1^. With the improvement of computed tomography screening technology, small lesions could be detected, making it possible for more patients in the early stage to be cured. However, there are still many patients who are already in a late stage when diagnosed and have missed the best surgical treatment period^2,3^. How to precisely treat this group of patients and improve their disease-free survival and overall survival is key to the diagnosis and treatment of advanced lung cancer^4^.

Patients with advanced lung cancer are often in poor physical condition and can hardly tolerate invasive examinations. This is because bleeding or other complications could easily occur during the procedure due to the large tumor loads. Therefore, it is of great benefit to patients to diagnose, classify (e.g. to distinguish between small cell and non-small cell carcinomas) and stage tumors through minimally invasive approaches, and this remains a popular research direction towards precision therapy^5,6^. Whilst histological biopsy and liquid-based cytological test (LCT) are both minimally invasive approaches for diagnosing lung cancer, the latter is even less invasive, since the specimens are harvested through sputum, pleural effusion, endobronchial ultrasound-guided transbronchial needle aspiration, bronchoscopy brush examination, bronchoalveolar lavage fluid, etc.^7,8^

Pleural effusion commonly exhibits in advanced lung cancer patients, especially in those with lung adenocarcinoma, for whom LCT is often the first-line diagnostic test to determine the stage of tumor and to obtain a large number of tumor cells for further molecular examinations^9–11^. Although many patients with advanced lung cancer are diagnosed cytologically with pleural effusion aspiration in our daily practice, differentiating cancer cells from reactive mesothelial cells remains a challenging problem for pleural effusion cytological diagnosis^12^. Cell blocks and immunohistochemistry (IHC) are not suitable for every cytological specimen because of the limited amount of tissue; thus, cytology slides are often the only sample available for diagnosis. In these cases, cytological morphology is important. However, subjective observation of cytological morphology may result in low interobserver consistency, especially in confusing cases, even for senior pathologists^13^. Improving the discrimination of tumor cells and other cells can provide support for decision making in clinical practice and greatly reduce both physical and economic burdens^14–16^.

Artificial intelligence (AI) has been widely used in the field of modern medicine^17,18^ and can help pathologists make more accurate diagnoses^19–22^. Using a deep convolutional neural network (DCNN), AI can be used to establish a systematic method to evaluate cells and obtain a final result^23–26^. We focus on the applications of AI to cytology as the latter not only plays an important role in pathology but also has the potential to resolve many clinical problems^27^. The conventional predictive AI models used in decision support systems for medical image analysis rely on annotations and manually engineered feature extraction, which is time-consuming and requires the advanced skills of cytopathologists or experts^28,29^. However, the weakly supervised deep learning algorithm could solve the problem of mass annotation, thus not requiring any annotation but a label for samples to be used for the training and validation of the model^30–32^.

In this study, we performed a weakly supervised deep learning method (namely “Aitrox AI model”) for the classification of benign and malignant cases based on lung cytological images at the whole-slide image (WSI) level. To investigate the diagnostic performance of the Aitrox AI model, it was compared against that of junior cytopathologists (resident pathologists who studied thoracic cytology for less than 1 year) and senior cytopathologists (attending pathologists who independently handled cytological reports for more than 3 years) with the patient pathological reports used as the gold standard.

## Materials and methods

**Fig. 1:**
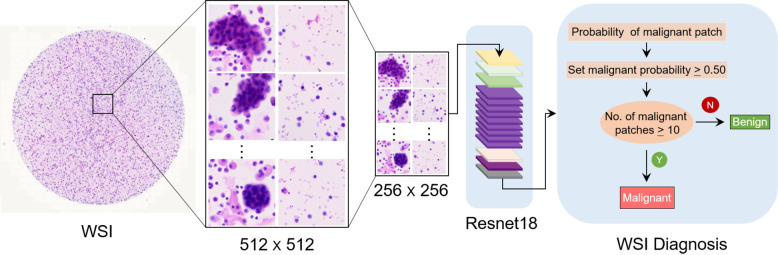
Overview of the proposed deep learning framework presented in this study. WSIs of lung cancer pleural effusion specimens were cropped into small patches and classified as benign or malignant lesions based on a Resnet18 deep convolutional neural network. WSI, whole-slide image.

This study was approved by the Institutional Ethics Committee of Shanghai Pulmonary Hospital (approval no. K19-127Y). The cell classification method proposed in this study is shown in Fig. 1. Patches were cropped from cytological WSIs, and the Aitrox AI model was then used to classify those patches as benign or malignant.

### Materials

In this study, we retrospectively reviewed patients with benign and pulmonary adenocarcinoma pleural effusion cases diagnosed by interventional fine-needle aspiration from March 2018 to January 2020 at Shanghai Pulmonary Hospital. For patients with multiple slides, only one slide with the highest quality was manually selected for downstream analysis. The final constructed dataset consisted of 234 benign and 170 malignant slides, among which 28% were diagnosed according to cytological morphology alone whereas 72% were diagnosed according to cytological morphology combined with a matched cell block with IHC. Clinical follow-ups were not included in these cases. All slides were checked by a senior cytopathologist (XFX) and stored in the online digital slide viewer Microscope Image Information System from Shanghai Aitrox Technology Corporation Limited, Shanghai, China. Cytological specimens were prepared by the LCT method and stained with hematoxylin and eosin (H&E). The LCT is a method used to prepare cytological slides using the characteristics of different types of human cells (cells with larger sizes are rapidly deposited on glass after sedimentation). In pleural effusion cytology, tumor cells are larger than normal cells, which makes them easily recognizable by pathologists. The procedure of cytology specimen preparation was conducted according to the manufacturer’s protocol (CytoRich Non-gyn, BD), but with a change involving staining with H&E as used in our daily practice.

Liquid-based cytology can greatly facilitate the digitization of pathology because during the sample preparation, mucus and red blood cells (which often appear in the traditional smear) are removed and other cells are displayed as a thin layer form on the slide. The flat and high-quality slices greatly improve the efficiency of WSI scanning^33^. The WSIs of 234 benign and 170 malignant slides were collected using a digital slide scanner with a 40× magnification objective and a resolution of 0.25 µm/pixel and saved in SVS format according to the manufacturer’s protocol (EasyScan 6, Motic Inc., Xiamen, China). Six slides were excluded due to difficulty in scanning during quality control, including those with out-of-focus blur.

### Data distribution

**Fig. 2:**
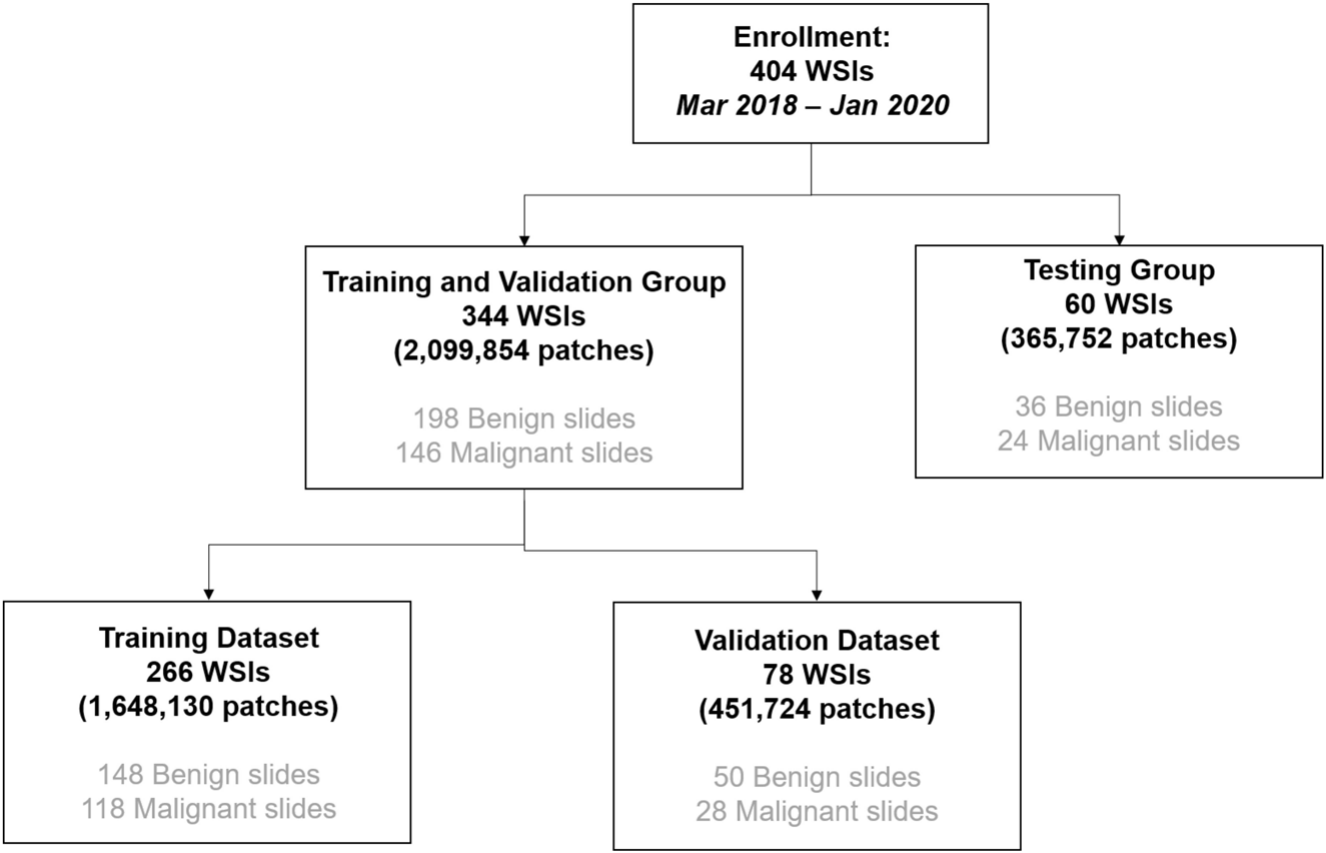
The consort diagram of the enrolled data from the Shanghai Pulmonary Hospital from March 2018 to January 2020. Enrolled 404 WSIs were randomly allocated into training (266 WSIs), validation (78 WSIs) and testing (60 WSIs) datasets. WSI, whole slide image.

A consort diagram for data enrollment and allocation is presented in Fig. 2. According to the malignancy distribution of the dataset, 404 WSIs were randomly allocated into training, validation and test sets to train the models, select the best model, and evaluate the performance of the best model^34,35^. Because the sizes of the WSIs were too large to directly input to a neural network, all WSIs were cropped into small patches. There were 266 slides with 1,648,130 patches in the training set, 78 slides with 451,724 patches in the validation set, and 60 slides with 365,752 patches in the test set. For model construction, 344 slides were split into two groups, namely, the training and validation groups, which optimized the accuracy of the AI model.

### Image preparation

**Fig. 3:**
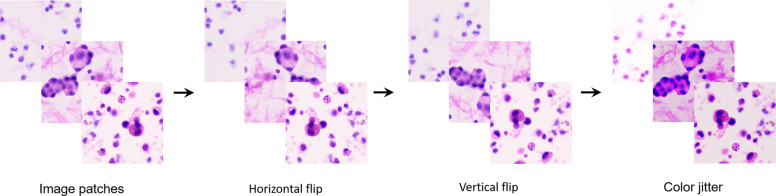
Generation and augmentation of patch images. Image patches were augmented by horizontal flip, vertical flip and color jitter.

To construct the image dataset for the DCNN, patch images of 512 × 512 pixels were first cropped from the original microscopic images without overlapping and then resized to 256 × 256 pixels as input for model training. The pathologists reviewed the WSIs, and the number of patches per slide depended on the sample size, with an average of 6104. Before data augmentation, 1,648,130 (754,622 malignant/893,508 benign) and 451,724 (178,392 malignant/273,332 benign) patch images were obtained for the training and validation sets, respectively. As shown in Fig. 3, the patch images included various kinds of cells, and data augmentation (such as random horizontal flipping, random vertical flipping, and color jitter) was introduced to generate more diverse images to improve the generalizability of our model. On average, there were 24,417 patches per slide after data augmentation. Finally, 6,592,520 (3,018,488 malignant/3,574,032 benign) and 1,806,896 (713,568 malignant/1,093,328 benign) augmented patch images were prepared for training and validation, respectively.

### DCNN model training

The weakly supervised WSI classification model relies on multiple instance learning (MIL). The slide-level diagnosis casts the same weak labels on all patches within a specified WSI: on the one hand, if the slide is classified as malignant, at least one patch image contains malignant cells; on the other hand, if the slide is classified as benign, all of its patches must be benign and completely free from malignant cells^31^. The ResNet18 network structure, which was first proposed by Kaiming He, was employed to construct the classification models in this study^36^. A two-step approach was adopted during model training. In the first stage, a classifier was trained with patch images and the corresponding weak labels. The weak label of a single patch image was defined as the label of the WSI within the patch. All patches in the training dataset were inferred by the pretrained classifier, of which the pretrained weights were obtained from pretraining on the ImageNet dataset^37^. The malignant probabilities of the patches inferred within the same slide were sorted from high to low. The top ten patches with the highest probabilities were selected for inclusion in the training set, and each patch obtained a weak label from their common WSI. In the traditional MIL method, *n* (the number of patches with the highest probabilities) is usually set to 1. However, since the number of malignant cells in the WSIs of pleural effusion was usually greater than 10, *n* was set to 10 to obtain more training data in this research, making the model more convergent and preventing overfitting. In addition, to balance the distribution between malignant and benign samples, ten additional patches from each negative WSI were randomly selected for inclusion in the training set. In the second stage, the classifier was updated with the training data obtained from the first stage.

## Resultsten

This study aimed to identify malignant and benign samples. Table 1 shows the baseline characteristics of the patients in all three datasets. The training and test sets achieved a balance in most of the characteristics. The p-values between the training and test sets for age and sex were 0.1307 and 0.3837, respectively, which are greater than 0.05 and indicate a balanced distribution between the two sets.

**Table 1:** Baseline characteristics of the patients in training, validation and testing groups.

Characteristics	Total (*n* = 404)	Training (*n* = 266)	Validation (*n* = 78)	Test (*n* = 60)	*P* value
Age, median (IQR), yr	66 (21–97)	65 (21–69)	65 (21–91)	65 (22–97)	0.1307
Male, No. (%)	265 (65.27%)	174 (65.41%)	54 (69.23%)	37 (59.68%)	0.3837
Histopathologic & Clinical final diagnosis					
Malignant lesions	170 (41.87%)	118 (44.36%)	28 (35.90%)	24 (38.71%)	
Benign lesions	234 (58.13%)	148 (55.64%)	50 (64.10%)	36 (61.29%)	

**Fig. 4:**
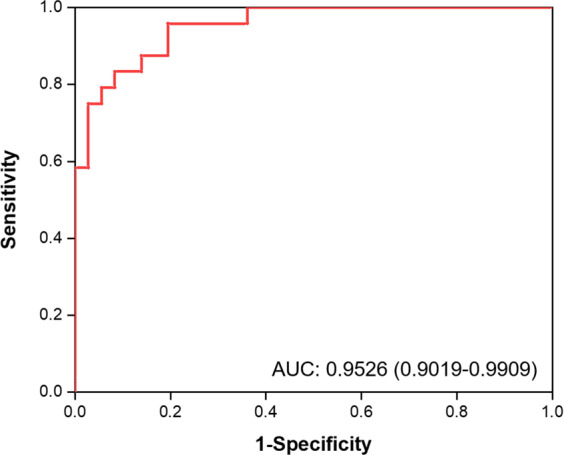
Receiver operator characteristics (ROC) curve of the AI model with AUC of 0.9526 (95% CI: 0.9019–0.9909). AUC denotes the area under the receiver operator characteristics curve.

The model had an area under the receiver operating characteristic curve (AUC) of 0.9526 with a 95% confidence interval (CI) of 0.9019–0.9909 (Fig. 4). If a patch has a malignancy probability greater than 0.50, it is considered a malignant patch. If a specific WSI has more than 10 malignant patches, it is classified as a malignant slide (Fig. 1).

The case-based classifier’s performance was tested on 60 slides and showed 91.67% accuracy, 87.50% sensitivity, and 94.44% specificity. The performance was compared with that of two senior and junior cytopathologists. The cytopathologists annotated the dataset at the WSI level and provided a binary result (malignant or benign) for each WSI. All given outputs were evaluated against the gold standard (the pathological report result). The accuracy of our AI model was 91.67%, with an AUC of 0.9526 (95% CI: 0.9019–0.9909). In comparison, the accuracies of the two senior cytopathologists (P1 and P2; P2 was an expert cytopathologist) and the two junior cytopathologists (P3 and P4) were 96.67%, 100.00%, 81.67%, and 85.00%, respectively.

**Fig. 5:**
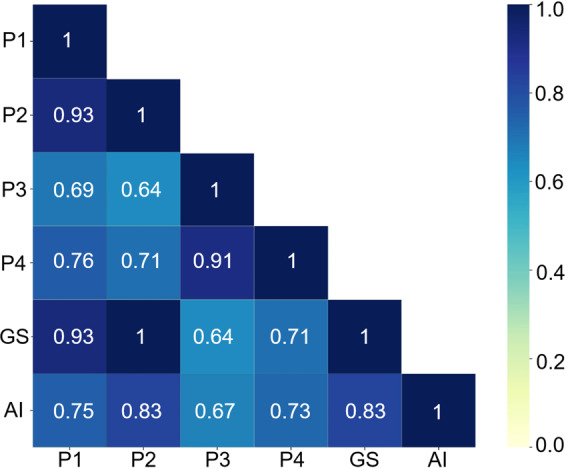
Heatmap of Kendall correlation *τ* value for gold standard, two senior cytopathologists, two junior cytopathologists and DCNN model. GS, Gold standard; P1 and P2, two senior pathologists; P3 and P4, two junior pathologists; AI, the Aitrox AI model based on a DCNN.

Correlations of the gold standard with the senior cytopathologists, junior cytopathologists, and Aitrox AI model were respectively analyzed. The results showed that the diagnoses by the senior pathologists (P1 and P2, Fig. 5) were most significantly correlated with the gold standard, with *τ*-values of 0.93 and 1.00. The diagnoses by the junior pathologists (P3 and P4, Fig. 5) showed the lowest correlation with the gold standard, with τ-values of 0.64 and 0.71. The predictions of the AI model were significantly correlated with the gold standard, with a *τ*-value of 0.83. The AI predictions also correlated with the diagnoses by the senior and junior cytopathologists, with *τ* values ranging from 0.67 and 0.83. The accuracy of the test set showed that the performance of our Aitrox AI model was between that of the junior and senior cytopathologists (Fig. 5).

**Fig. 6:**
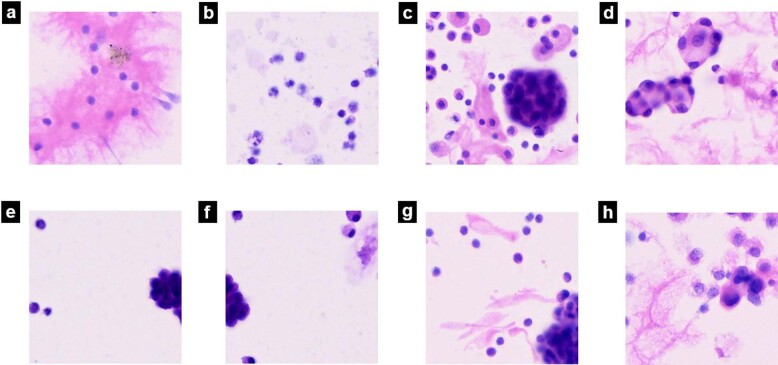
Different situation of AI performance in pleura effusion cytology diagnosis. **a** and **b** show AI made correct diagnosis of benign cells; **c** and **d** show AI made correct diagnosis of malignant cells; **e** and **f** show AI misdiagnosed benign mesothelial cells as malignant; **g** and **h** show AI misdiagnosed malignant cells as benign.

Figure 6 shows example patches from malignant and benign WSIs. Fig 6a–d and Fig. 6e–h show correct classifications and misclassifications by our DCNN, respectively.

## Discussion

To the best of our knowledge, this is the first study with the largest dataset that applied weakly supervised AI to cytological pleural effusion image evaluation and compared predictions from AI with diagnoses by cytopathologists at different WSI levels. Previous studies were conducted with a small amount of data at the patch level or single-cell level. For example, Win’s experiments on cytological pleural effusion were carried out on 125^38^ and 124^39^ cytological pleural effusion images. The cytological specimens of 46 patients were collected in Teramoto’s study on lung cytological images^40^. In Tosun’s study of mesothelial cells in effusion cytology specimens, only 34 patients were enrolled^12^. Compared with these studies, our investigation was carried out with a larger dataset of 404 patients, which indicated that our dataset had broader coverage and higher generalization.

The main purpose of this investigation was to evaluate whether diagnoses made by the weakly supervised Aitrox AI model could reach clinical grade at the WSI level. This was the first time that a weakly supervised deep learning method was introduced to model training to distinguish malignant cells in effusion cytology specimens. To achieve this goal, a deep learning method was developed based on the Resnet 18 structure adopting the MIL approach. Diagnoses were also made by senior cytopathologists, junior cytopathologists and the Aitrox AI model for the same test set comprising 60 WSIs. Kendall’s correlation coefficients between the Aitrox AI model and the cytopathologists and gold standard demonstrated that the Aitrox AI model predictions had strong correlations with the diagnoses by all cytopathologists (from 0.67 to 0.83) and the gold standard (0.83)^41^. The diagnoses by the senior cytopathologists showed the highest correlation with the gold standard (0.93 and 1.00), while those by the junior cytopathologists showed the lowest correlation with the gold standard (0.64 and 0.71), indicating that the Aitrox AI model has the potential to make clinical-grade decisions during the initial diagnosis.

Among the 60 WSIs in the test set, 5 WSIs were misclassified by our AI model. The AI model performed well in classifying images with straightforward cell morphology and obvious features (Fig. 6a–d). However, the presence of proliferating mesothelial cells that cluster together (Fig. 6e, f) and tumor cells with poor adhesion (Fig. 6g, h) may result in misclassification by our DCNN. Of note, the diagnoses made in Fig. 6e and f are also difficult for pathologists in real clinical scenarios, and further evaluation based on cell blocks with IHC by experienced pathologists is necessary.

Cytological diagnosis plays an important role in the rapid diagnosis of lung cancer. Since cytological diagnosis is very subjective and requires experience, it takes a long time to train cytopathologists. Both the severe shortage of cytopathologists and the low accuracy rate of junior cytopathologists limit the applications of cytological diagnosis in China. Our research shows that the AI model has an accuracy rate close to that of senior cytopathologists, which may compensate for the shortage of experienced cytopathologists through its deployment in different hospitals and cities.

In the present study, we explored the performance of a DCNN model in the classification of cancer cells on cytological pleural effusion images at the WSI level. The results showed that the AI model achieved a diagnostic accuracy of 91.67%, which was much better than that of the two junior cytopathologists. The results indicated that AI may assist pathologists in diagnosing cancer cells on cytological pleural effusion images in the future.

This study has some limitations. Our data were collected from a single medical center (Shanghai Pulmonary Hospital), and only lung adenocarcinoma patients were included. While lung adenocarcinoma represents the most common subtype of lung cancer, future studies would benefit from including additional samples of other lung cancer types from multiple national and international centers. Another potential limitation is the exclusion of any atypical or suspicious diagnoses. A possible direction for future studies to follow up will be developing a DCNN-based method that can not only classify malignant and benign lesions but also output the malignant probability of a WSI to better assist pathologists in diagnosing atypical or suspicious cases.

## Data Availability

The datasets used and/or analyzed during the current study are available from the corresponding author on reasonable request.
